# Saving the frontline health workforce amidst the COVID-19 crisis: Challenges and recommendations

**DOI:** 10.7189/jogh-10-010345

**Published:** 2020-06

**Authors:** Shubha Nagesh, Stuti Chakraborty

**Affiliations:** 1 Latika Roy Foundation, Dehradun, Uttarakhand, India; 2 Christian Medical College and Hospital, Ida Scudder Road, Vellore, Tamil Nadu, India

Healthcare workers are toiling countless number of hours since the global outbreak of COVID-19 (caused by the SARS CoV-2 and also known as the novel coronavirus) which has been recently declared as a pandemic by the World Health Organisation [1]. Healthcare systems across developed and developing nations are being put to the ultimate test and are under tremendous pressure to limit the spread of the novel coronavirus and majority of this responsibility is being shouldered by frontline health care workers effortlessly putting their lives on the line in order to do so. Through this article, we attempt to highlight some of the dire challenges currently being faced by frontline health workers and propose certain recommendations to reduce the encumbrance being imposed on them in order to ensure the provision of rapid, well-equipped, efficient health care services.

Unconditional and unprejudiced servitude is one of the principles health workers around the world adhere to, especially in time of peril as the present day. The last two weeks have been unusual and have set the norm for a new-normal- it is an understatement to state that the COVID-19, apart from causing widespread morbidity, mortality and fear, has specifically thrown the health care workforce on emergency mode. Reports from affected countries have revealed in the past that 22% of health care workers were affected in hospitals across Hong Kong, with the initial wave managing to infect 80% of the staff working in the medical wards of Prince Wales Hospital. This is just one example of the magnitude with which this virus has taken a toll on the health workforce [2].

Reported for the first time in Hanoi, Vietnam, Severe Acute Respiratory Distress Syndrome or SARS became known to us as far back as 2003. Globally, history seems to repeat itself, as is evident from the figures released by the National Health Commission of China, with more than 3300 health care professionals infected in March, causing at least 46 deaths as of 17 March 2020. Italy reported 20% of their health care workforce being infected, with many losing their lives to the virus [3,4].

The exposure to the virus causes debility, morbidity and mortality - but to a significant extent, also leads to immense physical and psychological exhaustion. This breakdown has led to health departments and ministries reaching out to retired medical staff and those who spend more time in research etc, to return to clinical work, to tide over the mounting pressures on the health system. As is evident in the last week, from all over the world, hospitals and other health facilities are reporting deficient supplies of personal protective equipment and other vital necessities. Those working in the emergency and the intensive care units are particularly and disproportionately affected, when they are the ones who need it the most. In addition to battling endless hours, draining shifts, staff shortages and deficient supplies, most are isolated from their families, affecting them emotionally and physically. Anxiety, depression, denial, insomnia and anger in the face of this pandemic will take a toll on the health service delivery adding to the increasing morbidity and ill health. These mental health problems are not only affecting health workers’ clinical decision-making ability, judgement, attention and understanding of the disease but might also have a lasting impact on their overall well-being [5].

Perhaps the largest misery remains the shortages in personal protective equipment for frontline health care workers, who are now resorting to reuse single use gear or develop local, generic quick fixes, both of which do not offer the same protection as professional quality gear. In Wuhan, the epicentre of the outbreak, medical professionals have reported using tape to patch up torn masks, reused one-time use eye goggles, and wrap their toes in domestic use plastic bags, for lack of availability of shoe covers. It has been worse for female frontline health care workers who have reportedly had to shave their heads to prevent spread of the virus, and are not being provided necessary sanitary hygiene services to take care of their menstrual needs [6,7].

The first official report of a health professional who succumbed to the virus is Dr Li Wenliang, 34, an ophthalmologist, who not only attempted to warn the world of this imminent threat, but also battled the bureaucracy as he battled the virus. Many other ministers and health leaders have since been infected or died, as they led national and regional efforts to combat this rapidly progressing virus [8,9].

Meanwhile in India (country of residence of the authors), there have been 8447 cases and 273 deaths (as of 12 April 2020, 17:00 hours), while several continue to believe that community transmission has set in. Experts forecast the trajectory of India to follow that of China, given the commonalities in terms of vast populations, crowded settlements and other factors. Epidemiologists are projecting that limited testing (52 laboratories to be specific) for a densely populated country, with a very robust travel history, could be a fatal mistake on part of the decision-making authorities. A bigger cause of concern is of course the deficient infrastructure with only 1.3 beds per 1000 population in hospitals, in comparison to WHO projections of 3.5 beds per 1000 population [10-12].

Since the first case was reported in the southern state of Kerala, many cases have now been detected from all over the country, and at a pace more rapid than expected. Most of the initial cases did not disclose travel history on time, and went on to infect multiple health care providers, all of whom and their families are in quarantine now. With each of these crucial professionals at home, the facilities become deficient in capacity, exponentially, if not more. In a country already deficient in staff, supplies, beds and ventilators, unnecessary recklessness on behalf of patients, leads to further losses in capacity and therefore management [13].

**Figure:**
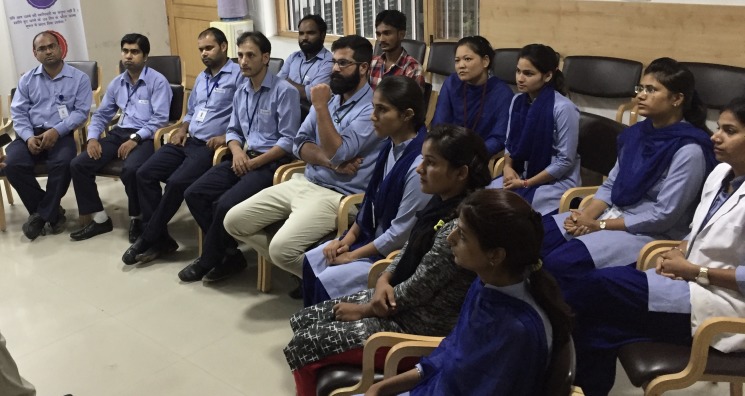
Photo: Image credit and copyright: Dr. Shubha Nagesh (used with permission).

Proposed below are a few recommendations which have surfaced from all the information received around this issue which must not be overlooked:

1) **Test frontline health care staff as a priority** – Much more than hospitals, equipment and face masks, health care staff are the most important resource in present times. Epidemics and outbreaks stretch limits in other ways- many health care workers report Post Traumatic Stress Disorder, and are alert with no time to protect themselves and their families. As staff fall sick and are quarantined themselves, shortages begin to show and healthy staff are stretched further, all leading to a vicious cycle of endless duties, lack of sleep, anxiety and maybe errors, eventually. Testing kits must be prioritized mainly for frontline health workers, vulnerable communities more susceptible to the virus and those with pre-existing comorbidities [14].

2) **Providing PPE to all frontline workers to protect themselves** – Health workers face a dire risk of contracting the disease themselves as they provide care, given the high infectivity of the virus. Protective clothing, gloves, eye glasses, sufficient hand sanitizers, washing paraphernalia, gowns and head covers are essential commodities that health workers should be provided in adequate measure. Dissemination of PPEs can be leveraged based on risk-assessments to high-contact/ transmission areas. Regular monitoring, surveillance and follow-up can delay complete shortage. Apart from providing PPEs, adequate disposal methodology must also be reinforced across all clinical areas.

3) **Self-isolation, social isolation and quarantine,** are key phrases many are talking about, will help to break the chain of transmission and allow systems the time to gather resources and capacity. It is quite clear that the virus infects exponentially and many will contract it very soon. States must think of innovative means to open up premises to serve as isolation and quarantine spaces. All hospitals must be utilized in their full capacities to create dedicated hospital control committees that can monitor activities, conduct surveillance and ensure protocols are implemented for effective control. Involving community systems, governments and primary health care workers is key, if the loop has to be complete, since community transmission will be rampant and not everyone will report to hospitals.

4) **Utilisation of training, knowledge and protocols to follow** – The comfort in working for a health system that is prepared and has an effective plan is magnified many times over in a pandemic. Algorithms for triage, protocols in local languages, and awareness material based on with evidence from science and research will be useful. Offering free shuttle services between work and home, childcare support and meal vouchers for staff could also reduce domestic stress and allow for a single-minded effort towards health service provision.

## CONCLUSION

Effective, rapid responsive leadership, clarity in pandemic guidelines and collaborations with primary health care, all encapsulated in systematic implementation, based on evidence and science, is essential to beat this outbreak. Keeping the aims of Universal Health Coverage (UHC) in mind, it is essential to act timely, yet decisively in this regard as this pandemic has challenged us with many “novels”, than just the virus per se.
